# Levofloxacin-induced Psychosis in a Young Healthy Patient

**DOI:** 10.7759/cureus.6217

**Published:** 2019-11-22

**Authors:** Michael Muradian, Shaheena Khan

**Affiliations:** 1 Department of Internal Medicine, Medical College of Wisconsin, Milwaukee, USA

**Keywords:** levofloxacin, acute psychosis, neurotoxicity, medication side effects

## Abstract

Levofloxacin is a fluoroquinolone antibiotic commonly used to treat a wide range of bacterial infections. It is normally well tolerated with the most common side effect being gastrointestinal distress. Severe side effects including neuropsychiatric symptoms are rare but have been observed even in patients who lack risk factors or are otherwise healthy. Healthcare providers should be aware of these effects and consider transitioning to other antibiotics in patients who develop psychosis or other neuropsychiatric symptoms after initiating this medication. Symptoms rapidly improve after discontinuation of the drug, so prompt recognition can reduce morbidity and mortality. We report a case of levofloxacin-induced psychosis in a young healthy female patient.

## Introduction

While commonly used for their favorable characteristics and their activity against a range of bacteria, fluoroquinolones can have severe side effects. While most commonly patients can have mild symptoms such as gastrointestinal distress, severe neuropsychiatric effects can occur. These effects, which are highly variable, are rare and not well described in the literature. Here we describe a case of levofloxacin induced psychosis in a 23-year-old female being treated for bilateral submandibular abscesses.

## Case presentation

A 23-year-old African American female with a history of recurrent submandibular abscesses and a submental abscess currently being treated with levofloxacin and metronidazole presented to the hospital emergency department with anxiety, paranoia, visual hallucinations, and psychosis. She had first been diagnosed with a dental abscess approximately one month prior and was prescribed clindamycin due to allergies to antibiotics including amoxicillin, ampicillin, and penicillin. Due to issues with medication non-adherence the patient subsequently developed bilateral submandibular abscesses and a submental abscess requiring repeat incision and drainage and transition to alternative antibiotics. She had been recently discharged from the hospital four days prior following repeat incision and drainage with the prescriptions for levofloxacin and metronidazole, which she had been taking as instructed. At outpatient follow-up two days after discharge, she reported feeling well and appeared to be clinically improving; she had no symptoms at that time. One day later she developed progressive paranoia and insomnia, and the following day she developed visual hallucinations described as dark shapes. She was then brought to the emergency department by family. Examination was unremarkable with the exception of the psychiatric examination; she showed a flat affect, poor eye contact, and had fluent speech. She appeared internally preoccupied with her hallucinations, and her responses to questions were somewhat limited. According to her family, she had no personal or family history of psychiatric illness and had never had these symptoms previously. Labs on admission included a drug and toxin screen which was negative and a head CT which showed no signs of intracranial pathologies. Other labs showed no evidence of metabolic causes for altered mental status such as hyponatremia, hypernatremia, other electrolyte abnormalities, hypoglycemia, or encephalopathies. Given her lack of psychiatric history and that her symptoms were not consistent with any primary psychiatric disorder or other organic cause, a concern was raised that the condition may have been caused by the antibiotics. The levofloxacin and metronidazole were promptly discontinued, and she was then transitioned to ceftriaxone. Over the following days, additional testing including an electroencephalogram was negative and no other causes of psychosis could be identified. She gradually improved and reported decreased paranoia, insomnia, and the absence of persistent hallucinations. Three days after discontinuing the antibiotics, she had returned to baseline mental status per the patient and her family. She was then transitioned to oral cefdinir and discharged to home with instructions to follow up with her surgeon and with psychiatry.

## Discussion

Levofloxacin is an antibiotic of the fluoroquinolone drug class that exerts their effect by inhibiting bacterial DNA gyrase [1]. It is generally well tolerated with a favorable side effect profile, and it has activity against gram-positive bacteria, gram-negative bacteria, and *Pseudomonas* making it a commonly prescribed antibiotic [2]. Adverse drug reactions have been seen in approximately 2% of patients with gastrointestinal distress being the most common [3]. Neuropsychiatric side effects are very rare and poorly described in the literature with psychosis only being reported in approximately one in six million prescriptions [3]. It is believed that this effect may be related to possible inhibition of GABA receptors by levofloxacin [1,4]. Additionally, prior reports have suggested that renal disease may increase the risk for psychiatric effects and many patients had other significant comorbidities [4,5]. The time of symptom onset in relation to the initiation of antibiotic treatment is highly variable which makes identifying the cause more complicated. One prior report documented symptoms hours after beginning treatment, while in other cases it has taken several days [1,4,5]. Antibiotic-induced psychosis has been observed to resolve within a few days of drug discontinuation in prior reports [5]. While rare, these effects of levofloxacin are severe and reversible. This case highlights how otherwise healthy individuals with no significant risk factors or psychiatric history are susceptible. Medical providers should consider these poorly understood effects in any patient receiving this drug who develops psychiatric symptoms.

## Conclusions

Antibiotics including levofloxacin can be a cause of acute psychosis in patients even if they lack risk factors. While neuropsychiatric side effects are rare, they can be severe; levofloxacin is a commonly used antibiotic and providers should be aware of this possible effect. Medical providers should maintain a high index of suspicion when there is a temporal relationship between the initiation of the drug and symptom onset. As this case highlights, discontinuation of the medication can lead to rapid resolution of symptoms and early recognition is important to reduce long-term morbidity.

## References

[1] Moorthy N, Raghavendra N, Venkatarathnamma PN (2008). Levofloxacin-induced acute psychosis. Indian J Psychiatry.

[2] Steuber H, Williams D, Rech M (2018). Leave the levofloxacin? A case report of levofloxacin-induced psychosis. Am J Emerg Med.

[3] Carbon C (2001). Comparison of the side effects of levofloxacin versus other fluoroquinolones. Chemotherapy.

[4] Takser L, Grand R (2017). Acute psychotic symptoms following a single dose of levofloxacin. Clin Case Rep.

[5] Lertxundi U, Palacios RH, Gutierrez FC, Domingo-Echaburu S, Garcia GG, Gomez CA (2013). Levofloxacin-induced delirium in a patient suffering from schizoaffective disorder and multiple sclerosis. Curr Drug Saf.

